# Supplementary material for: Pre-stimulus activity mediates event-related theta synchronization and alpha desynchronization during memory formation in healthy aging
Source: Imaging Neurosci (Camb). 2024 Dec 16;2:imag-2-00398. doi: 10.1162/imag_a_00398 (PMC12315746; doi:10.1162/imag_a_00398)
Supplement: Supplementary Material [file imag_a_00398-suppl.pdf]

1. Supplementary Material

1.1. Reaction times

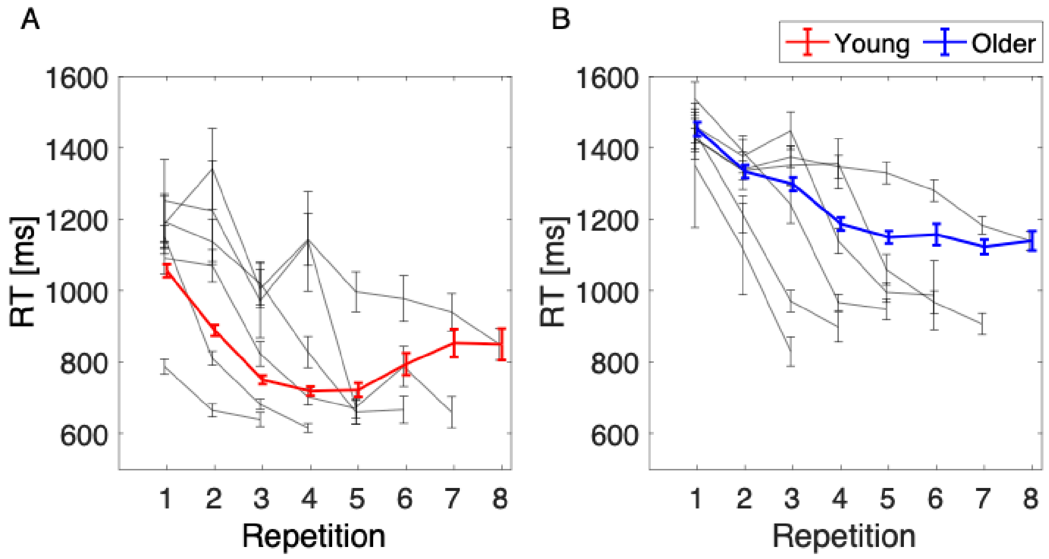

Supplementary Figure 1: Reaction times in young (A) and older (B) participants. Please note that the participants were not explicitly instructed to perform the task as fast as possible. Hence, the reaction times need to be interpreted with caution.

1.2. Grand average time-frequency representation

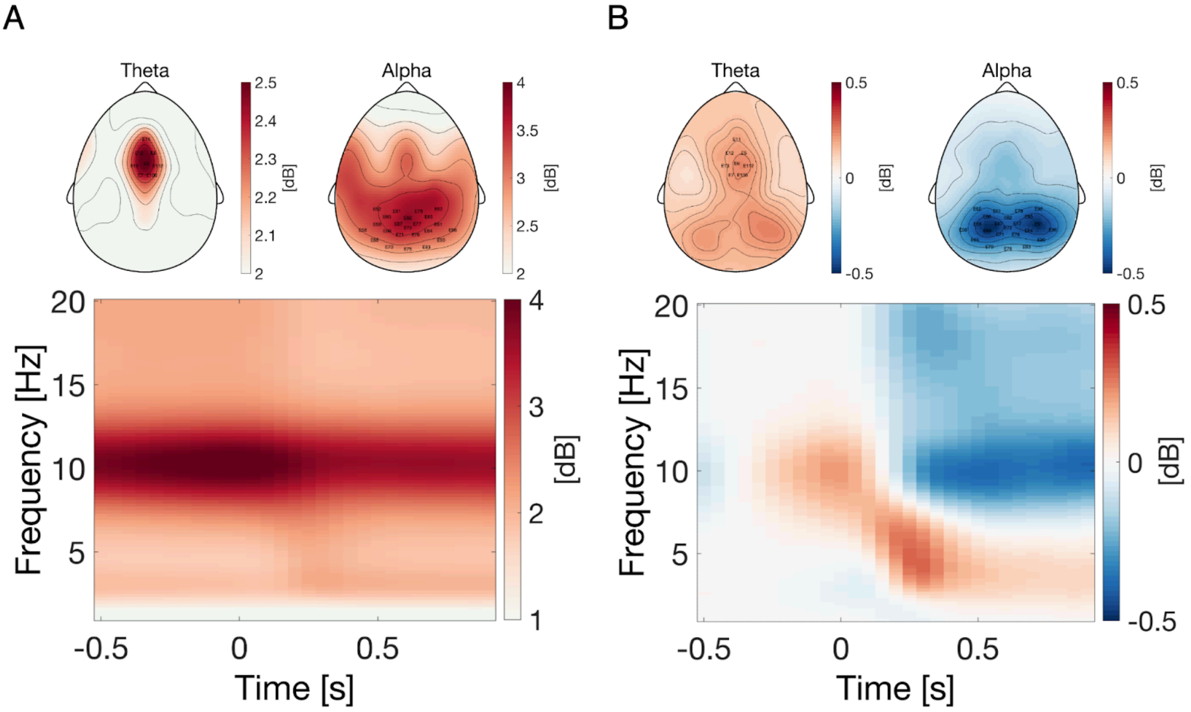

Supplementary Figure 2: Grand average time-frequency representation (TFR) over all age groups, sequence repetitions and channels. (A) Aperiodic adjusted total power. (B) Change in aperiodic-adjusted power relative to pre-stimulus baseline (i.e., -500 to -250 ms). Scalp topographies

were averaged over time windows spanning 100 to 500 ms for mid-frontal theta, and 250 to 900 ms for parietal alpha. Highlighted electrodes were used for further statistical analysis.

1.3. Conventionally analyzed ERS & ERD over sequence repetition

In the main manuscript, we employed the specParam algorithm to disentangle periodic and aperiodic components from the EEG signal. As a control analysis and to confirm previous ERS & ERD findings, we also conducted the analysis using conventionally computed power, without the removal of aperiodic components. We followed the same procedure and data analysis steps as in section XX up to aperiodic slope removal. We started the analysis by computing the same model on theta ERS that we previously applied after the removal of aperiodic component.

ThetaERS ~ RepetitionNr * AgeGroup + (1 | ID) + (1 | SequenceNr)

The model revealed a significant main effect of repetition number ($\beta = -0.20$; $p = 1.96e{-16}$; CI = [-0.25, -0.15]), indicating a decrease of mid-frontal theta ERS with each sequence repetition. Furthermore, there was a significant main effect of age group ($\beta = -0.27$; $p = 0.009$; CI = [-0.48, -0.07]), that is, the mid-frontal theta ERS was decreased in older compared to young subjects. There was also a significant interaction of repetition number and age group ($\beta = 0.16$; $p = 1.36e{-8}$; CI = [0.10, 0.21]), indicating a more gradual decrease of mid-frontal theta ERS across sequence repetitions in older subjects (Supplementary Table 1).

Supplementary Table 1. Effects of repetition number and age group on mid-frontal theta ERS

Variable	***β***	***SE***	***CI***	***t-value***	***p-value***
Intercept	1.36	0.08	1.20 – 1.52	16.28	2.26e-41***
RepetitionNr	-0.20	0.02	-0.25 – -0.15	-8.25	1.96e-16***
AgeGroup [Old]	-0.27	0.10	-0.48 – -0.07	-2.62	0.009**
RepetitionNr*AgeGroup [Old]	0.16	0.03	0.10 – 0.21	5.69	1.36e-8***
**Variance components**	**SD**	**Goodness of fit**			
Subject	0.41	Log likelihood		-10561.5	
SequenceNr	0.05				
Residual	1.49				

Note. Intercept represents the first repetition of young participants. RepetitionNr = Repetition number. β = unstandardized regression coefficient. SE = Standard error. CI = Confidence interval. SD = Standard deviation.
$*p < 0.05$. $**p < 0.01$. $***p < 0.001$.

Next, we proceeded to conduct a similar analysis for parietal alpha ERD. Again, we applied the same statistical model as described in the main manuscript.

AlphaERD ~ RepetitionNr * AgeGroup + (1 | ID)

This model revealed a significant main effect of repetition number (β = -0.11; p = 2.99e-11; CI = [-0.14, -0.08]), indicating a greater parietal alpha ERD with each sequence repetition. The main effect of age group (β = -0.40; p = 5.82e-7; CI = [-0.56, -0.25]) was statistically significant, providing evidence for age-related differences in overall parietal alpha ERD aggregated across repetitions between young and older subjects. Furthermore, there was a significant interaction of repetition number and age group (β = 0.10; p = 1.07e-7; CI = [0.06, 0.14]), reflecting a more gradual increase of parietal alpha ERD across sequence repetitions in older subjects (Supplementary Table 2). Summarized, the conventionally computed power revealed a significant main effect of the age group. In contrast, when we analyzed the power with the aperiodic component removed, this age-related effect was not observed. This absence indicates that the influence of age on EEG measurements might predominantly reside within the aperiodic components of the signal, rather than the periodic components.

Supplementary Table 2. Effects of repetition number and age group on parietal alpha ERD

Variable	*β*	*SE*	*CI*	*t-value*	*p-value*
Intercept	0.24	0.06	0.12 – 0.36	4.02	6.32e-5***
RepetitionNr	-0.11	0.02	-0.14 – -0.08	-6.66	2.99e-11***
AgeGroup [Old]	-0.40	0.08	-0.56 – -0.25	-5.04	5.82e-7***
RepetitionNr*AgeGroup [Old]	0.10	0.02	0.06 – 0.14	5.32	1.07e-7***
**Variance components**	**SD**	**Goodness of fit**			
Subject	0.38	Log likelihood		-8414.25	
Residual	1.02				

Note. Intercept represents the first repetition of young participants. RepetitionNr = Repetition number. β = unstandardized regression coefficient. SE = Standard error. CI = Confidence interval. SD = Standard deviation.
**p < 0.05. **p < 0.01. ***p < 0.001.*

1.4. ERS & ERD over learning categories

So far, we demonstrated that the mid-frontal theta ERS decrease and parietal alpha ERD increase with sequence repetitions. However, the modulations of mid-frontal theta and parietal alpha could be simply an effect of habituation and the power could decrease or increase as a function of time spent on a task. Therefore, to provide stronger evidence for the relationship of mid-frontal theta ERS and parietal alpha ERD with learning, we tested whether the modulations of both components change as a function of learning state, that is, from stimulus being unknown, to newly learned, known and finally very well known, regardless of the sequence repetition number.

First, each individual stimulus presentation was classified as one of five categories based on the responses of the participants after each sequence repetition. A stimulus was assigned to the *unknown* category (UN) when location was recalled incorrectly in the current and all previous repetitions, to the *newly learned* category (NL) when stimulus position was recalled correctly for the first time in the current repetition, to the *known* category (K) when stimulus position was correctly recalled at least twice in a row, to the *very well known* category (K+) when stimulus position was correctly recalled at least three times in a row, and to the *forgotten* category (F) when stimulus position was once recalled correctly but incorrectly in the following sequence repetition. The *forgotten* category was removed from further analysis due to the low number of trials. Next, we identified the best-fit model for the mid-frontal theta

ERS and parietal alpha ERD. Because we were merely interested in power differences across learning categories and age groups, we additionally computed contrasts for those variables using the *emmeans* package in R Studio [80]. Supplementary Figure 3 shows the time-frequency decomposition of young and older subjects averaged over all 105 electrodes as a function of the learning category.

Mid-frontal theta

$$\text{ThetaERS} \sim \text{Category} * \text{AgeGroup} + (1 \mid \text{ID})$$

The model revealed a significant main effect of category K+ (β = -0.19; p =0.010 ; CI = [-0.33, -0.05]), indicating decreased mid-frontal theta ERS for K+ compared to UN (i.e., intercept) trials. The other effects did not reach significance (Supplementary Table 3). Next, we proceeded with computing contrasts between learning categories and age groups. The results of contrast comparisons are summarized in Supplementary Table 4.

Supplementary Table 3. Effects of learning category and age group on mid-frontal theta ERS

Variable	***β***	***SE***	***CI***	***t-value***	***p-value***
Intercept	0.34	0.07	0.21 – 0.46	5.14	2.81e-7***
NL	0.12	0.07	-0.02 – 0.26	1.63	0.103
K	-0.07	0.07	-0.21 – 0.07	-0.95	0.342
K+	-0.19	0.07	-0.33 – -0.05	-2.60	0.010*
AgeGroup [Old]	-0.12	0.08	-0.27 – 0.04	-1.49	0.136
NL*AgeGroup [Old]	-0.11	0.09	-0.29 – 0.07	-1.16	0.247
K*AgeGroup [Old]	0.06	0.09	-0.13 – 0.24	0.60	0.550
K+*AgeGroup [Old]	0.12	0.09	-0.05 – 0.31	1.45	0.147
**Variance components**	**SD**	**Goodness of fit**			
Subject	0.17	Log likelihood		-4636.92	
Residual	0.82				

Note. Intercept represents the first repetition of young participants. NL = newly learned, K = known, K+ = better known. β = unstandardized regression coefficient. SE = Standard error. CI = Confidence interval. SD = Standard deviation.
**p < 0.05. **p < 0.01. ***p < 0.001.*

In young participants, the contrast comparison revealed a slightly larger mid-frontal theta ERS for UN trials relative to K ($\beta = 0.07$; $p = 0.999$; CI = [-0.07, 0.21]) and K+ ($\beta =$; $p =$; CI = []) trials, however the differences did not reach statistical significance. Mid-frontal Theta ERS for NL trials was slightly larger than UN trials ($\beta = -0.11$; $p = 0.927$; CI = [-0.25, 0.02]), but this was not statistically significant. NL trials showed significantly larger mid-frontal theta ERS compared to K ($\beta = 0.18$; $p = 0.001$; CI = [0.09, 0.28]) and K+ ($\beta = 0.30$; $p = 1e\text{-}10$; CI = [0.21, 0.40]) trials. Finally, K trials exhibited greater mid-frontal theta ERS than K+ trials, but the difference was not statistically significant.

In older subjects, we observed trends similar to those seen in the younger cohort. The mid-frontal theta ERS in UN trials appeared slightly larger when compared to both K ($\beta = 0.01$; $p = 0.999$; CI = [-0.10, 0.13]) and K+ ($\beta = 0.05$; $p = 0.999$; CI = [-0.05, 0.16]) trials. Additionally, NL trials seemed to exhibit a marginally higher mid-frontal theta ERS compared to UN ($\beta = -0.01$; $p = 0.999$; CI = [-0.12, 0.10]) trials. Finally, K trials exhibited greater mid-frontal theta ERS than K+ ($\beta = 0.04$; $p = 0.999$; CI = [-0.04, 0.17]) trials. These trends mirrored the patterns we noted in young participants. However, none of these comparisons in the older group reached statistical significance.

When comparing both age groups, young showed a higher mid-frontal theta ERS in UN trials compared to their older counterparts ($\beta = 0.12$; $p = 0.999$; CI = [-0.03, 0.27]). However, this difference was not statistically significant. Notably, the most pronounced difference was observed in NL trials ($\beta = 0.29$; $p = 1e\text{-}6$; CI = [0.18, 0.40]), where young subjects exhibited significantly larger mid-frontal theta ERS than older subjects. Conversely, while young participants also displayed slightly larger mid-frontal theta ERS in K trials ($\beta = 0.06$; $p = 0.999$; CI = [-0.05, 0.17]), this difference did not reach statistical significance. Finally, in K+ trials, young subjects had marginally lower mid-frontal theta ERS compared to older subjects, but again, this was not statistically significant ($\beta = -0.01$; $p = 0.999$; CI = [-0.12, 0.09]).

Supplementary Table 4. Mid-frontal theta ERS: contrast comparisons between age groups and learning categories.

**Contrast**	***β***	***SE***	***CI***	***z-ratio***	***p-value***
UN Young - NL Young	-0.11	0.07	-0.25 – 0.02	-1.63	0.927
UN Young - K Young	0.07	0.07	-0.07 – 0.21	0.95	0.999
UN Young - K+ Young	0.18	0.07	0.05 – 0.33	2.60	0.085
NL Young - K Young	0.18	0.05	0.09 – 0.28	3.85	0.001**
NL Young - K+ Young	0.30	0.05	0.21 – 0.40	6.35	1e-10***
K Young - K+ Young	0.11	0.05	0.02 – 0.21	2.45	0.129
UN Old - NL Old	-0.01	0.05	-0.12 – 0.10	0.86	0.999
UN Old - K Old	0.01	0.06	-0.10 – 0.13	0.22	0.999
UN Old - K+ Old	0.05	0.06	-0.05 – 0.16	0.98	0.999
NL Old - K Old	0.02	0.06	-0.08 – 0.13	0.43	0.999
NL Old - K+ Old	0.06	0.05	-0.04 – 0.17	1.23	0.999
K Old - K+ Old	0.04	0.05	-0.04 – 0.17	0.22	0.999
UN Young - UN Old	0.12	0.08	-0.04 – 0.27	1.49	0.999
NL Young - NL Old	0.29	0.05	0.18 – 0.40	5.35	1e-6***
K Young - K Old	0.06	0.06	-0.05 – 0.17	1.12	0.999
K+ Young - K+ Old	-0.01	0.05	-0.12 – 0.09	-0.23	0.999

Note. UN = Unknown. NL = Newly learned. K = Known, K+ = Better known. β = unstandardized regression coefficient. SE = Standard error. CI = Confidence interval. P-values adjusted using the Bonferroni method for 16 tests.
$*p < 0.05$. $**p < 0.01$. $***p < 0.001$.

Parietal alpha

The model revealed a significant main effect of category K+ (β = -0.44; p = 3e-4; CI = [-0.65, -0.23]), indicating increased parietal alpha ERD for K+ compared to UN (i.e., intercept) trials. Moreover, there was a significant interaction of the K+ trials and age group (β = 0.43; p = 0.001; CI = [0.17, 0.69]), that is older subjects had decreased parietal alpha ERD for K+ trials than young subjects. The other effects did not reach significance (Supplementary Table 5). Next, we proceeded with computing contrasts between learning categories and age groups. The results of contrast comparisons are summarized in Supplementary Table 6.

Supplementary Table 5. Effects of learning category and age group on parietal alpha ERD

Variable	***β***	***SE***	***CI***	***t-value***	***p-value***
Intercept	-0.10	0.11	-0.31 – 0.12	-0.86	0.392
NL	-0.15	0.11	-0.36 – 0.06	-1.39	0.163
K	-0.18	0.11	-0.39 – 0.03	-1.66	0.097
K+	-0.44	0.11	-0.65 – -0.23	-4.15	3e-4***
AgeGroup [Old]	-0.19	0.14	-0.47 – 0.08	-1.37	0.170
NL*AgeGroup [Old]	0.00	0.14	-0.27 – 0.27	0.01	0.993
K*AgeGroup [Old]	0.08	0.14	-0.19 – 0.35	0.58	0.561
K+*AgeGroup [Old]	0.43	0.13	0.17 – 0.69	3.20	0.001**
**Variance components**	**SD**	**Goodness of fit**			
Subject	0.64	Log likelihood		-6196.78	
Residual	1.20				

Note. Intercept represents the first repetition of young participants. NL = newly learned, K = known, K+ = better known. β = unstandardized regression coefficient. SE = Standard error. CI = Confidence interval. SD = Standard deviation.
**p < 0.05. **p < 0.01. ***p < 0.001.*

In young participants, the contrast comparison revealed smaller parietal alpha ERD for UN trials relative to NL (β = 0.15; p = 0.999; CI = [-0.06, 0.36]), K (β = 0.18; p = 0.868; CI = [-0.03, 0.39]) and K+ (β = 0.44; p = 2e-4; CI = [0.23, 0.65]) trials, however the difference between UN and K, and UN and NL trials did not reach statistical significance. NL trials exhibited a slightly smaller parietal alpha ERD compared to K trials, though the difference was not statistically significant (β = 0.03; p = 0.999; CI = [-0.11, 0.17]). In contrast, NL trials demonstrated a significantly smaller parietal alpha ERD than K+ (β = 0.29; p = 3e-4; CI = [0.15, 0.43]) trials. Finally, K trials exhibited significantly greater smaller ERD than K+ trials (β = 0.26; p =0.001; CI = [0.13, 0.40]). Summarized, parietal alpha ERD increased as the knowledge strengthened.

Unlike in younger participants where a trend was observed in parietal alpha ERD as knowledge increased, older subjects did not show any significant trends across different learning categories. UN trials showed smaller parietal alpha ERD compared to NL (β = 0.15; p = 0.747; CI = [-0.02, 0.31]), K (β = 0.10; p = 0.999; CI = [-0.07, 0.26]), and K+ (β = 0.01; p = 0.999; CI = [-0.15, 0.17]) trials. However, none of these differences reached statistical

significance. NL trials exhibited larger parietal alpha ERD compared to both K (β = -0.05; p = 0.999; CI = [-0.21, 0.11]) and K+ (β = -0.14; p = 0.729; CI = [-0.29, 0.02]) trials, but these differences were not statistically significant. Similarly, K trials had larger parietal alpha ERD compared to K+ (β = -0.09; p = 0.999; CI = [-0.24, 0.06]) trials, yet this difference was also not statistically significant.

Finally, we compared both age groups. The parietal alpha ERD was smaller in young participants compared to older participants in UN (β = 0.19; p = 0.999; CI = [-0.08, 0.47]), NL (β = 0.19; p = 0.828; CI = [-0.03, 0.42]) and K (β = 0.12; p = 0.999; CI = [-0.11, 0.34]) trials. However, none of these effects reached statistical significance. Interestingly, the pattern reversed in K+ trials, where older participants demonstrated smaller, yet not significant parietal alpha ERD compared to young participants (β = -0.23; p = 0.329; CI = [-0.45, -0.01]).

Supplementary Table 6. Parietal alpha ERD: contrast comparisons between age groups and learning categories.

**Contrast**	***β***	***SE***	***CI***	***z-ratio***	***p-value***
UN Young - NL Young	0.15	0.11	-0.06 – 0.36	1.39	0.999
UN Young - K Young	0.18	0.11	-0.03 – 0.39	1.66	0.868
UN Young - K+ Young	0.44	0.11	0.23 – 0.65	4.15	2e-4***
NL Young - K Young	0.03	0.07	-0.11 – 0.17	0.41	0.999
NL Young - K+ Young	0.29	0.07	0.15 – 0.43	4.18	3e-4***
K Young - K+ Young	0.26	0.07	0.13 – 0.40	3.74	0.001**
UN Old - NL Old	0.15	0.08	-0.02 – 0.31	1.73	0.747
UN Old - K Old	0.10	0.08	-0.07 – 0.26	1.15	0.999
UN Old - K+ Old	0.01	0.08	-0.15 – 0.17	0.15	0.999
NL Old - K Old	-0.05	0.08	-0.21 – 0.11	-0.61	0.999
NL Old - K+ Old	-0.14	0.08	-0.29 – 0.02	-1.75	0.729
K Old - K+ Old	-0.09	0.08	-0.24 – 0.06	-1.11	0.999
UN Young - UN Old	0.19	0.14	-0.08 – 0.47	1.37	0.999
NL Young - NL Old	0.19	0.11	-0.03 – 0.42	1.69	0.828
K Young - K Old	0.12	0.12	-0.11 – 0.34	1.00	0.999
K+ Young - K+ Old	-0.23	0.11	-0.45 – -0.01	-2.09	0.329

Note. UN = Unknown. NL = Newly learned. K = Known, K+ = Better known. β = unstandardized regression coefficient. SE = Standard error. CI = Confidence interval. P-values adjusted using the

Bonferroni method for 16 tests.
**p < 0.05. **p < 0.01. ***p < 0.001.*

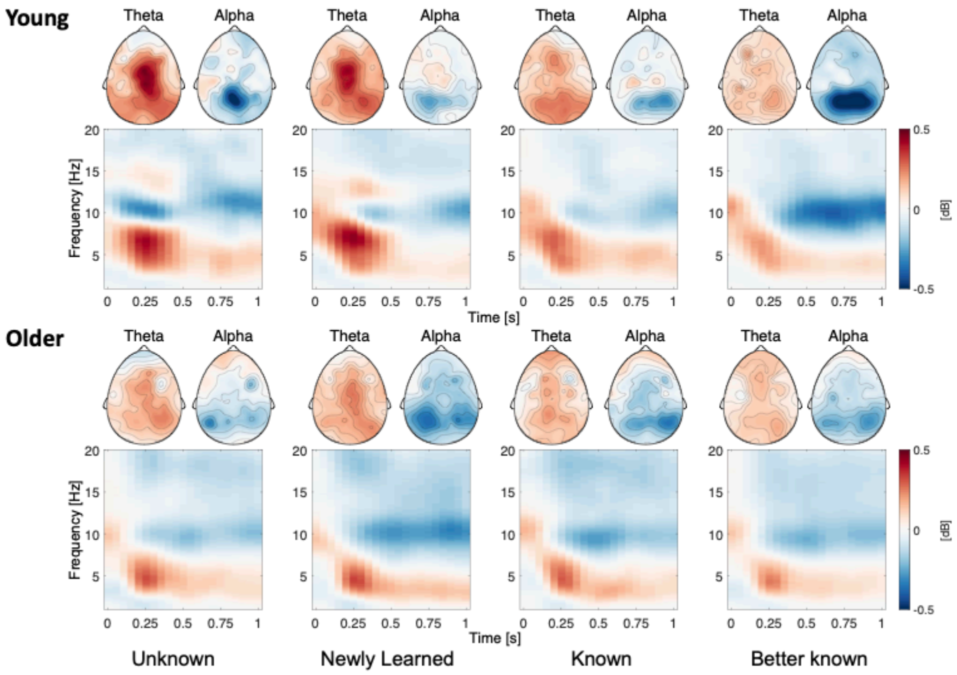

Supplementary Figure 3: Scalp topographies and time-frequency representation (TFR) of the ERS & ERD over learning categories in young (top) and older (bottom) subjects. The TFR spectrogram was averaged across all electrodes. Mid-frontal theta and parietal alpha topographies were averaged according to IAF. Blue colors signal event-related desynchronization (ERD), red colors signal event-related synchronization (ERS).

In summary, mid-frontal theta ERS and parietal alpha ERD exhibit distinct trajectories during the learning process. Parietal alpha ERD displayed a continuous decrease, whereas mid-frontal theta ERS peaked at the point of first accurate recall (i.e., newly learned). Notably, both age groups demonstrated similar patterns in these signals. However, the modulations of mid-frontal theta and parietal alpha were more pronounced in the younger group.

1.5. Finer temporal analysis: distance to first accurate recall

To further substantiate the connection between mid-frontal theta ERS, parietal alpha ERD, and the process of learning, we aligned the sequence repetitions to the point where the entire sequence was correctly recalled for the first time (i.e., considering the number of repetitions before or after this point). The first entire correctly recalled sequence was labeled as distance 0, with previous repetitions assigned negative distances and subsequent ones

positive distances. This approach allowed for a more detailed investigation of the learning process over time, offering finer temporal resolution than our earlier learning state categorizations (Supplementary Figure 4).

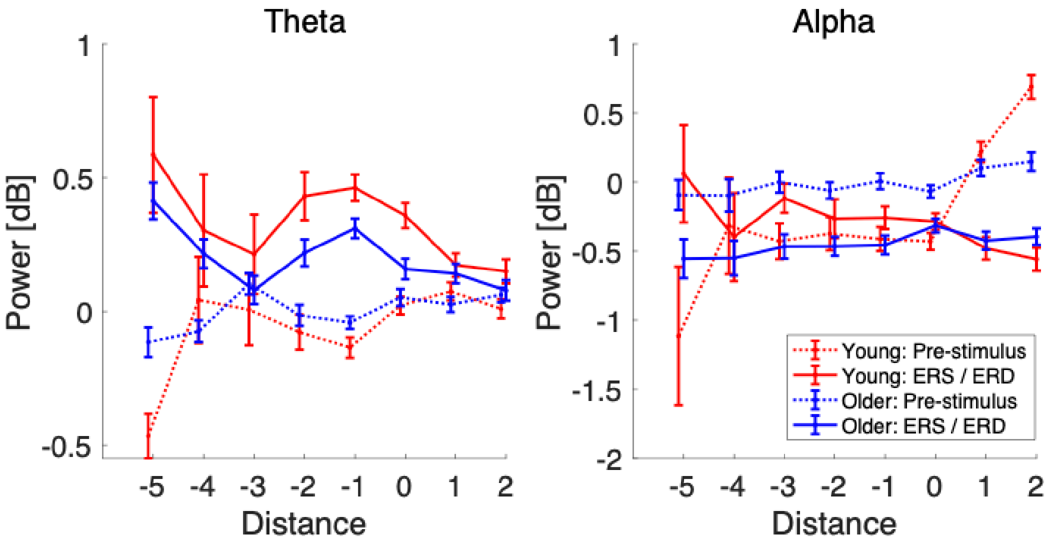

Supplementary Figure 4: Prestimulus power, mid-frontal theta ERS and parietal alpha ERD aligned to the point of first fully accurate recall (i.e., distance of 0). Please note that some of the subjects memorized the entire sequence within just three repetitions (i.e., distances 0, 1, 2), thus completing the task early. This led to a decrease in the number of subjects, and consequently the data, contributing to distances below zero and above two. Error bars represent the standard error of the mean.

Supplementary Table 7. Effects of distance and age group on mid-frontal theta ERS

Variable	***β***	***SE***	***CI***	***t-value***	***p-value***
Intercept	0.31	0.03	0.26 – 0.36	11.75	3.95e-25***
Distance	-0.06	0.01	-0.08 – -0.04	-5.35	9.22e-8***
AgeGroup [Old]	-0.12	0.04	-0.19 – -0.05	-3.23	0.001**
Distance*AgeGroup [Old]	0.05	0.01	0.02 – 0.07	3.39	0.001**
**Variance components**	**SD**	**Goodness of fit**			
Subject	0.18	Log likelihood		-6280.21	
Residual	0.90				

Note. Intercept represents the distance ‘-5’ of young participants. β = unstandardized regression coefficient. SE = Standard error. CI = Confidence interval. SD = Standard deviation.

Supplementary Table 8. Effects of distance and age group on parietal alpha ERD

Variable	*β*	*SE*	*CI*	*t-value*	*p-value*
Intercept	-0.36	0.07	-0.49 – -0.23	-5.31	2.64e-7***
Distance	-0.06	0.02	-0.09 – -0.02	-3.24	0.001**
AgeGroup [Old]	-0.00	0.10	-0.19 – 0.19	-0.02	0.985
Distance*AgeGroup [Old]	0.06	0.02	0.02 – 0.11	2.88	0.004**
**Variance components**	**SD**	**Goodness of fit**			
Subject	0.63	Log likelihood		-8357.51	
Residual	1.37				

Note. Intercept represents the distance '-5' of young participants. β = unstandardized regression coefficient. SE = Standard error. CI = Confidence interval. SD = Standard deviation.

Supplementary Table 9. Effects of distance and age group pre-stimulus mid-frontal theta ERS

Variable	*β*	*SE*	*CI*	*t-value*	*p-value*
Intercept	0.00	0.00	-0.06 – 0.07	0.10	0.919
Distance	0.01	0.01	0.00 – 0.01	2.89	0.004**
**Variance components**	**SD**	**Goodness of fit**			
SequenceNr	0.08	Log likelihood		-5033.7	
Residual	0.70				

Note. Intercept represents the distance '-5' of young participants. SequenceNr = Sequence number. β = unstandardized regression coefficient. SE = Standard error. CI = Confidence interval. SD = Standard deviation.

Supplementary Table 10. Effects of distance and age group on pre-stimulus parietal alpha ERD

Variable	*β*	*SE*	*CI*	*t-value*	*p-value*
Intercept	-0.19	0.10	-0.40 – 0.01	-1.86	0.063
Distance	0.21	0.02	0.18 – 0.25	11.78	1.41e-31***
AgeGroup [Old]	0.17	0.05	0.07 – 0.26	3.48	4.97e-4***
ET	0.02	0.01	0.01 – 0.04	2.56	0.010*
Distance*AgeGroup [Old]	-0.17	0.02	-0.21 – -0.13	-7.62	2.97e-14***
**Variance components**	**SD**	**Goodness of fit**			
SequenceNr	0.23	Log likelihood		-8558.39	
Residual	1.48				

Note. Intercept represents the distance '-5' of young participants. SequenceNr = Sequence number. β = unstandardized regression coefficient. SE = Standard error. CI = Confidence interval. SD = Standard deviation.

1.6. Moderated mediation

To investigate the age-related variations in how repetition number influences mid-frontal theta ERS and parietal alpha ERD, and to understand the role of pre-stimulus activity in this relationship, we employed a moderated mediation analysis with the age group as a moderator.

Mid-frontal theta

Initially, we established a significant main effect of repetition number on mid-frontal theta ERS in young subjects ($\beta = -0.19$; $p = 2e-16$; $CI = [-0.25, -0.14]$), which demonstrated a decrease in mid-frontal theta ERS with increasing sequence repetition. Subsequently, our Model M indicated that pre-stimulus mid-frontal theta power in young subjects increased with sequence repetition ($\beta = 0.14$; $p = 3.81e-6$; $CI = [0.08, 0.20]$). However, these effects were found to be moderated by age group, as evidenced by significant interactions between repetition number and the age group. In the case of older subjects, the decrease in mid-frontal theta ERS over repetitions was less pronounced ($\beta = 0.16$; $p = 4.19e-6$; $CI = [0.09, 0.23]$), and there was a less substantial increase in pre-stimulus mid-frontal theta power ($\beta = -0.10$; $p = 0.005$; $CI = [-0.17, -0.03]$). The mediation analysis yielded a significant average causal mediation effect (ACME) of pre-stimulus mid-frontal theta power on mid-frontal theta ERS in young ($\beta = -0.08$; $p = 2e-16$; $CI = [-0.11, -0.04]$) and older ($\beta = -0.03$; $p = 0.002$; $CI = [-0.05, -0.01]$) subjects. An average direct effect (ADE; the effect of repetition number on mid-frontal theta ERS after considering the pre-stimulus mid-frontal theta power) remained significant in young ($\beta = -0.12$; $p = 2e-16$; $CI = [-0.17, -0.07]$), but not older ($\beta = 0.01$; $p = 0.202$; $CI = [-0.04, 0.01]$) subjects (Supplementary Figure 4).

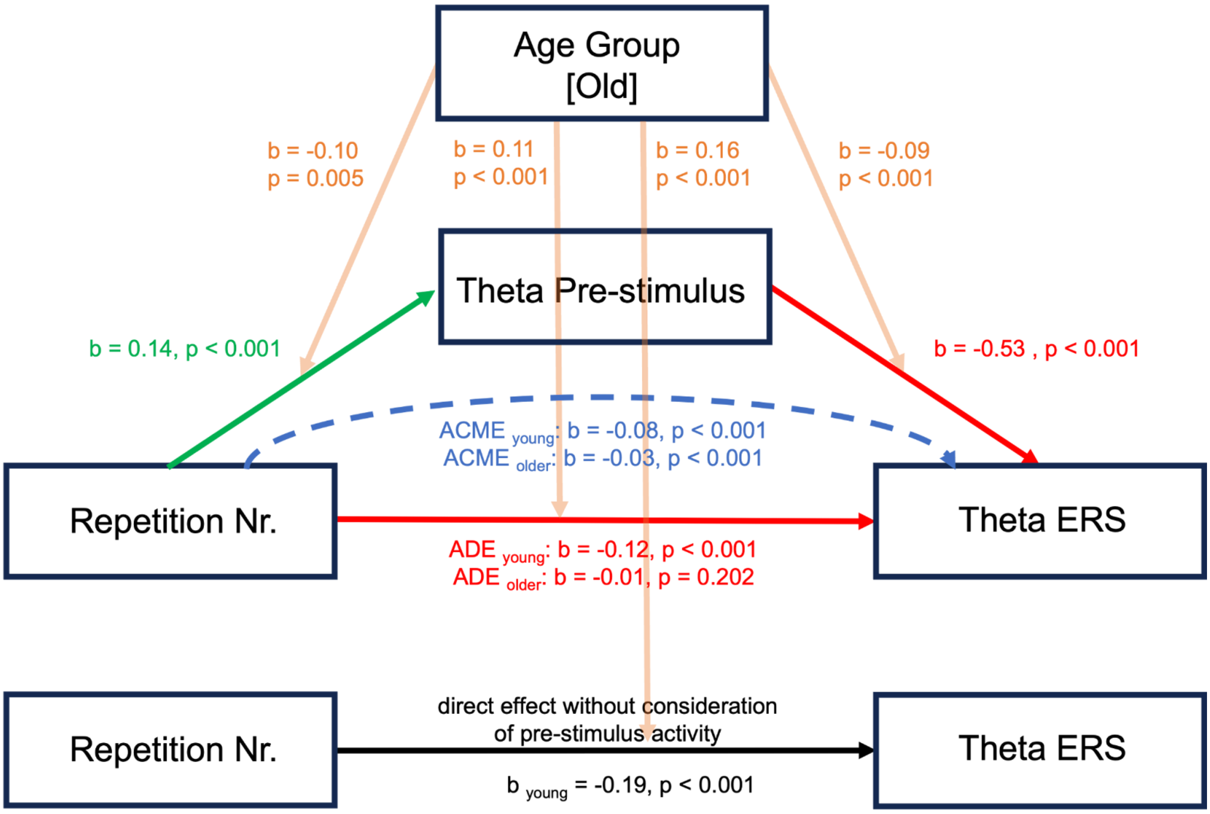

Supplementary Figure 4. Moderated mediation analysis investigating the effects of pre-stimulus mid-frontal theta power on mid-frontal theta ERS. The results show that a part of the total effect of repetition number on mid-frontal theta ERS is mediated by pre-stimulus mid-frontal theta power.

Parietal alpha

First, we confirmed a significant main effect of repetition number on parietal alpha ERD in young subjects ($\beta = -0.11$; $p = 2e\text{-}16$; CI = [-0.16, -0.06]), which indicated a greater parietal alpha ERD with an increase in sequence repetition. Subsequently, Model M demonstrated that pre-stimulus parietal alpha power in young subjects increased alongside sequence repetition ($\beta = 0.46$; $p = 4.64e\text{-}52$; CI = [0.40, 0.52]). However, these effects appeared to be moderated by age group, as shown by significant interactions between repetition number and the age group. For older subjects, the increase in parietal alpha ERD over repetitions was less pronounced ($\beta = 0.11$; $p = 4.94e\text{-}4$; CI = [0.05, 0.17]), and there was a more modest increase in pre-stimulus parietal alpha power ($\beta = -0.40$; $p = 1.51e\text{-}31$; CI = [-0.47, -0.33]). The mediation analysis yielded a significant average causal mediation effect (ACME) of pre-stimulus parietal alpha power on parietal alpha ERD in both young ($\beta = -0.28$; $p = 2e\text{-}16$; CI = [-0.32, -0.25]) and older ($\beta = -0.04$; $p = 2e\text{-}16$; CI = [-0.06, -0.02]) subjects. Moreover, an average direct effect (ADE; the effect of repetition number on parietal alpha ERD after considering pre-stimulus parietal alpha power) was significant in young subjects ($\beta = 0.17$;

p = 2e-16; CI = [0.13, 0.21]) and older subjects (β = 0.04; p = 0.002; CI = [0.01, 0.06]) (Supplementary Figure 5).

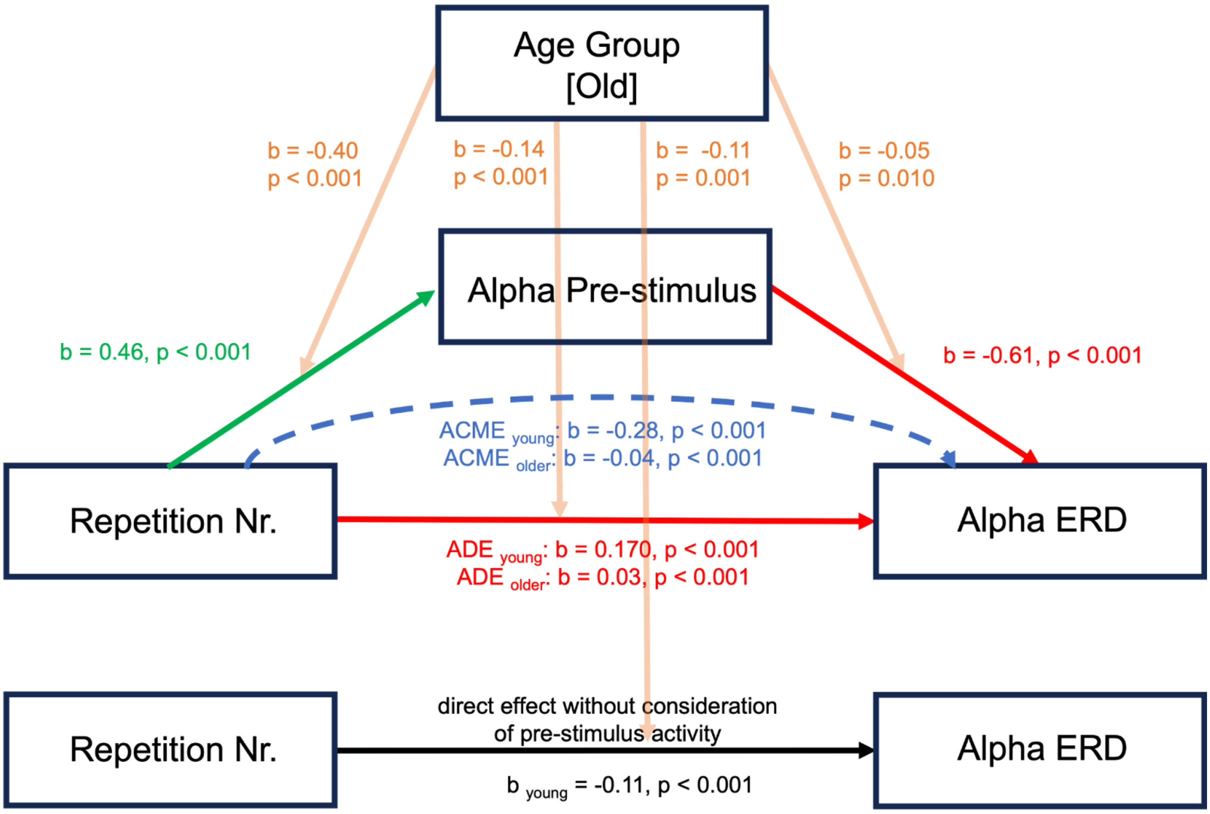

Supplementary Figure 5. Moderated mediation analysis investigating the effects of pre-stimulus parietal alpha power on parietal alpha ERD. The results show that a part of the total effect of repetition number on parietal alpha ERD is mediated by pre-stimulus parietal alpha power.

1. 7. Aperiodic component

While the aperiodic component was not the primary focus of this work, we provide a brief description of the results here for completeness. The detailed analysis and interpretation of the aperiodic signal remain outside the scope of this paper and are not included in the main text. Using the sliding window approach, we obtained aperiodic component estimates (i.e., aperiodic offset and exponent) ranging from -500 to 900 ms around stimulus onset, in 50 ms intervals, for each channel and sequence repetition. We next averaged the estimates over parieto-occipital electrode clusters (i.e., identical to parietal alpha electrodes) and plotted the progression of aperiodic offset and exponent over time and sequence repetitions (Supplementary Figure 6). The plot revealed very similar progressions of aperiodic offset and exponent, characterized by event-related increase of both signals shortly after stimulus

offset. Based on visual inspection, we fitted linear mixed-effects models to investigate pre-stimulus (-500 to -250 ms), event-related increase (0 to 500 ms), and late (500 to 900 ms) modulations of the aperiodic exponent over sequence repetitions and age groups (Supplementary Tables 11, 12 and 13). The models revealed that older subjects exhibit flatter exponents than their younger counterparts in all three time windows. Moreover, the models indicated a significant positive effect of repetition number on the aperiodic exponent in both the pre-stimulus and late time windows, suggesting an increasing aperiodic exponent over the course of learning. However, in both cases, the effect was weaker in older subjects, as indicated by a significant interaction between repetition number and age group.

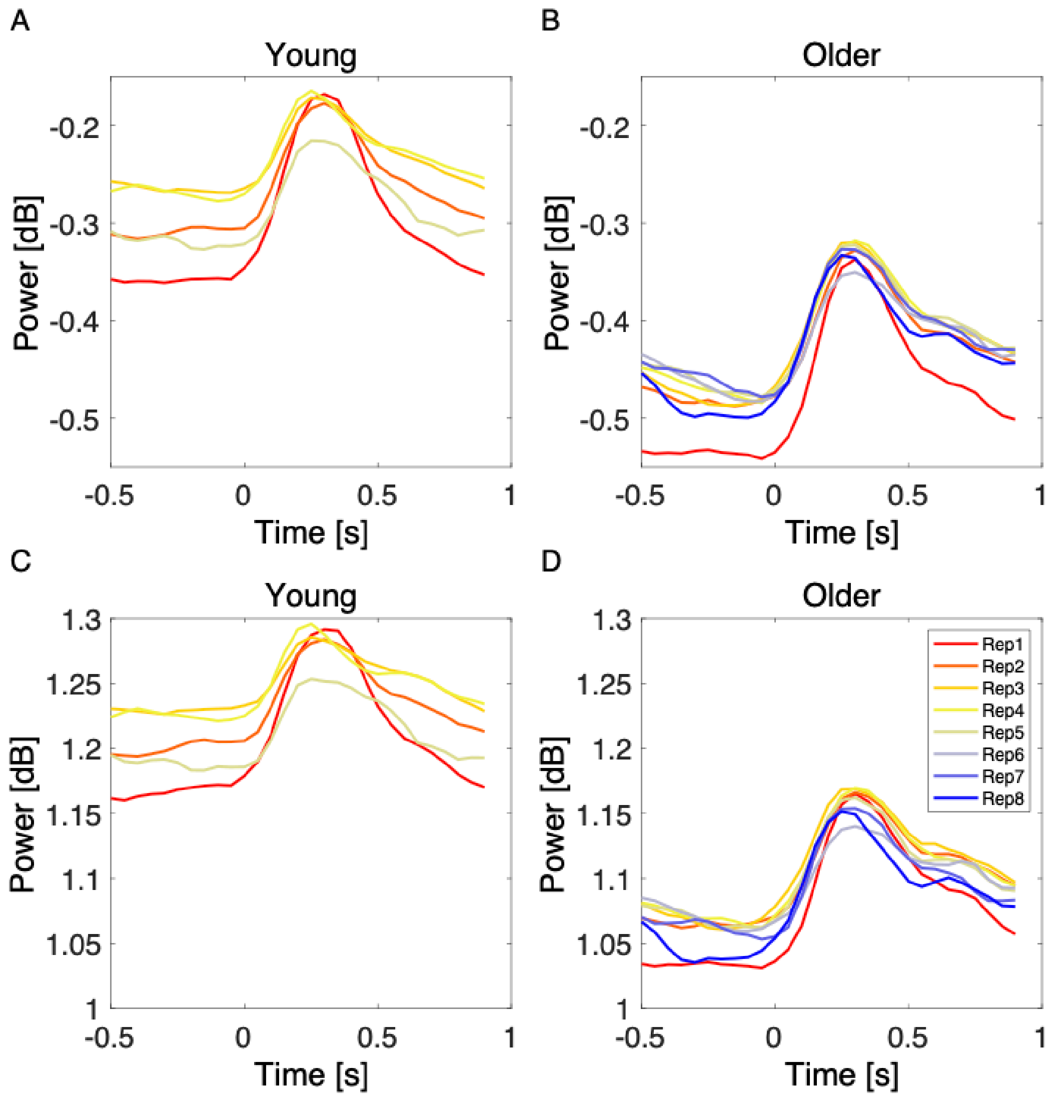

Supplementary Figure 6. Aperiodic offset (A-B) and exponent (B-C) over sequence repetitions in young and older subjects. On average, older subjects exhibited lower offset and flatter exponent compared to younger counterparts.

Supplementary Table 11. Effects of repetition number and age group on pre-stimulus aperiodic exponent

Variable	*β*	*SE*	*CI*	*t-value*	*p-value*
Intercept	-0.18	0.05	-0.27 – -0.08	-3.61	2.9e-4***
RepetitionNr	0.03	0.01	0.02 – 0.04	5.91	2.53e-9***
AgeGroup [Old]	-0.18	0.03	-0.24 – -0.12	-5.83	5.98e-9***
RepetitionNr*AgeGroup [Old]	-0.01	0.01	-0.01 – -0.00	-2.68	0.007**
**Variance components**	**SD**	**Goodness of fit**			
Subject	0.22	Log likelihood		1591.6	
Residual	0.17				

Note. Note. Intercept represents the first repetition of young participants. RepetitionNr = Repetition number. β = unstandardized regression coefficient. SE = Standard error. CI = Confidence interval. SD = Standard deviation.

Supplementary Table 12. Effects of repetition number and age group on event-related aperiodic exponent

Variable	*β*	*SE*	*CI*	*t-value*	*p-value*
Intercept	-0.05	0.05	-0.14 – 0.05	-1.04	0.298
RepetitionNr	0.00	0.00	0.00 – 0.02	1.21	0.225
AgeGroup [Old]	-0.19	0.03	-0.25 – -0.13	-6.05	1.57e-9***
RepetitionNr*AgeGroup [Old]	-0.00	0.00	-0.00 – 0.01	0.38	0.701
**Variance components**	**SD**	**Goodness of fit**			
Subject	0.22	Log likelihood		2254.8	
Residual	0.15				

Note. Note. Intercept represents the first repetition of young participants. RepetitionNr = Repetition number. β = unstandardized regression coefficient. SE = Standard error. CI = Confidence interval. SD = Standard deviation.

Supplementary Table 13. Effects of repetition number and age group on late aperiodic exponent

Variable	***β***	***SE***	***CI***	***t-value***	***p-value***
Intercept	-0.16	0.5	-0.26 – -0.06	-3.19	0.001**
RepetitionNr	0.02	0.00	0.01 – 0.03	5.59	2.41e-8***
AgeGroup [Old]	-0.16	0.03	-0.22 – -0.10	-5.00	5.76e-7***
RepetitionNr*AgeGroup [Old]	-0.01	0.00	-0.01 – -0.00	-2.80	0.005**
**Variance components**	**SD**	**Goodness of fit**			
Subject	0.22	Log likelihood		1912.2	
Residual	0.16				

Note. Note. Intercept represents the first repetition of young participants. RepetitionNr = Repetition number. β = unstandardized regression coefficient. SE = Standard error. CI = Confidence interval. SD = Standard deviation.

1. 8. Pre-stimulus activity over the entire inter-stimulus interval

Pre-stimulus activity could reflect not only predictive signals for the upcoming stimulus but also memory consolidation of the previous one(s). To further explore this alternative explanation, we conducted additional analyses to examine how neural activity changes across the entire inter-stimulus interval (ISI).

First, we visualized the modulations of mid-frontal theta and parietal alpha power over the entire trial duration (0 to 600 ms post-stimulus) and ISI (600 to 1900 ms post-stimulus) across sequence repetitions (Figure 1). Furthermore, we subdivided the ISI into an "early ISI" and a "late ISI" with the rationale that the early ISI would primarily reflect memory consolidation, while the late ISI would capture predictive signals for the upcoming stimuli. The early ISI was defined as 0.8 to 1.2 seconds post-stimulus, while the late ISI was defined as 1.3 to 1.9 seconds post-stimulus. These windows were chosen to avoid overlap with event-related activity and to capture the majority of the ISI, while accounting for the limited temporal resolution caused by the time-frequency trade-off inherent in time-frequency analyses.

For mid-frontal theta power, we observed across all repetitions a sharp increase shortly after stimulus presentation (event-related synchronization; ERS), which then returned to a baseline pre-stimulus level during the ISI. For parietal alpha power, we noted a general trend

of increasing power over sequence repetitions, with event-related desynchronization (ERD) following stimulus onset, and a subsequent rise before the next stimulus onset during the ISI. These patterns were consistent across both age groups.

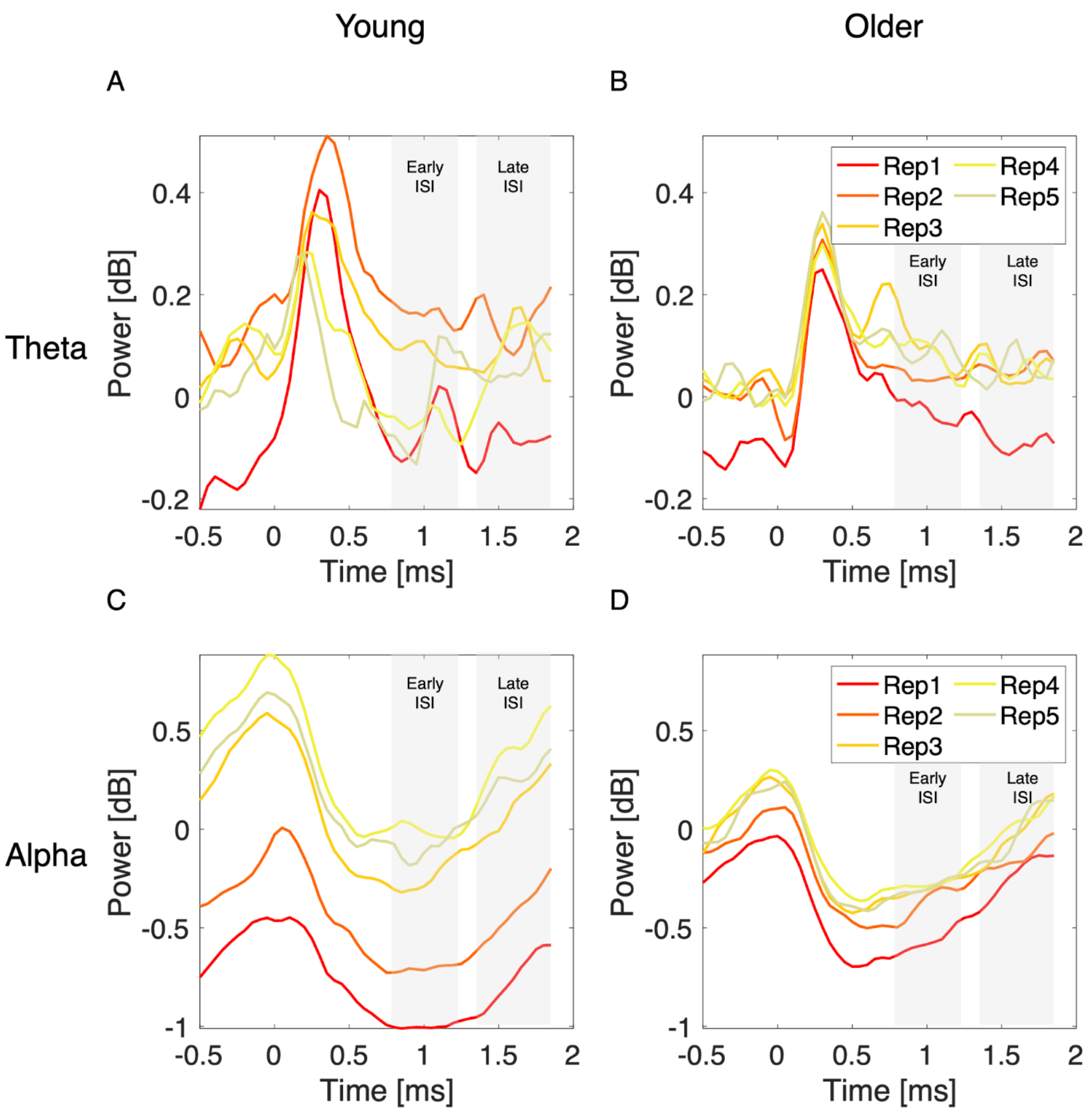

Supplementary Figure 7: Temporal evolution of mid-frontal theta (A & B) and parietal alpha power (C & D) over stimulus presentation period and inter-stimulus-interval in young (A & C) and older (B & D) participants across sequence repetitions.

Next, we compared the mid-frontal theta and parietal alpha power in the first and the last repetition of a sequence, where the last repetition was defined as the third correct recall of a given sequence. The rationale was that during the ISI of the first repetition, cognitive processes would be more focused on learning and consolidation rather than anticipation. This would manifest to an increase in mid-frontal theta activity and a prolonged parietal alpha ERD in the time interval immediately following the stimulus presentation (i.e., early

ISI). In contrast, cognitive processes during the last repetition would focus more on anticipation than on learning, as the sequence had already been correctly recalled twice. This would result in no increase in mid-frontal theta during the early ISI, with theta activity instead emerging in the late ISI, accompanied by a more rapid rise in parietal alpha throughout the entire ISI.

Indeed, as shown in Figure 2, we specifically observed in younger participants a second increase in theta power during the early ISI (around 0.8 to 1.2 second post-stimulus) of the first repetition, likely reflecting mental retrieval and consolidation. However, during the late ISI (around 1.3 to 1.9 seconds post-stimulus), mid-frontal theta power increased more in the last repetition compared to the first, likely reflecting anticipation and preparatory processes for the upcoming stimulus. This effect was not present in older participants, where mid-frontal theta power decreased gradually over the course of ISI in the first as well in the last sequence repetition.

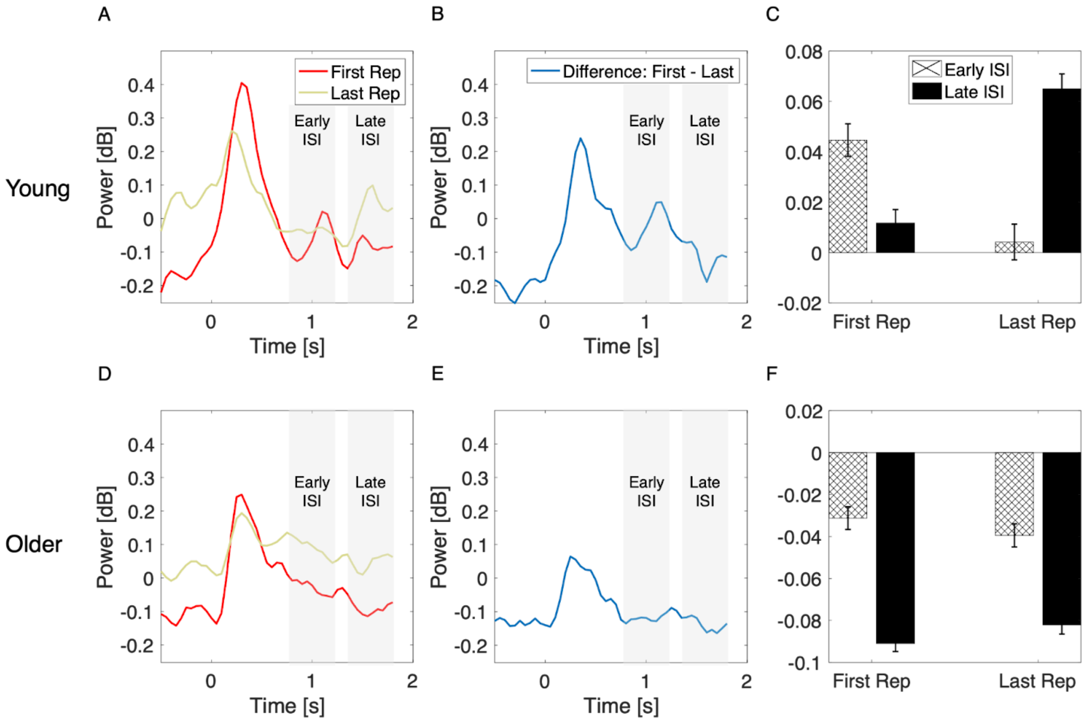

Supplementary Figure 8. Temporal evolution of mid-frontal theta power over stimulus presentation period and inter-stimulus-interval in young (A-C) and older (D-F) participants. Panels B and E show the difference between the first and the last (i.e., third correct recall for a given subject and sequence) sequence repetition. Panels C and F show modulation of power in early and late inter-stimulus-interval specific to ISI.

In panel C of Figure 2, we present the modulation of theta power by using the entire ISI interval as a reference. This approach allowed us to isolate and examine changes in mid-frontal theta power specific to the ISI, independent of event-related activity. Our analysis revealed that, in young participants, mid-frontal theta power exhibited a greater increase during the early ISI when comparing the first repetition to the last repetition. Conversely, in the late ISI, mid-frontal theta power exhibited a more pronounced increase during the last repetition compared to the first. It suggests distinct functional roles: enhanced mental retrieval and consolidation processes during the early ISI of the first repetition, and anticipatory and preparatory processes dominating the late ISI of the last repetition.

For parietal alpha power (Figure 3), we observed a general increase across sequence repetitions, as highlighted by the intercept in the difference wave panels in both age groups (Figure 3: B & E). Additionally, in younger participants, the parietal alpha ERD was greater during the last sequence repetition compared to the first, visible as an increase in power in the difference plot (Figure 3: B). However, from early ISI, this pattern reversed: during the first repetition, parietal alpha remained negative throughout the early ISI and increasing only in the late ISI. In contrast during the last repetition, alpha power began rising earlier, already in the early ISI, suggesting anticipation and preparation for the next stimulus (Figure 3: C). This indicates that in younger participants, during the early repetitions, when significant learning occurs, desynchronization persists longer (as seen by the difference in early ISI between first repetition and last repetition in Figure 3: C), likely due to mental retrieval and consolidation processes. This results in lower alpha power during both the early and late ISI compared to the last repetition. On the other hand, in the last repetition, with less new information to learn, the duration of alpha desynchronization shortens, and anticipation (manifested as increased alpha power) becomes more prominent. In older participants, these consolidation or retrieval processes seem less effective, as indicated by the absence of prolonged desynchronization in early repetitions. This might explain their lower behavioral performance in the task.

In summary, our findings suggest that pre-stimulus activity during the ISI likely reflects a combination of both memory consolidation for the previous stimulus and anticipatory mechanisms for the upcoming stimulus. However, given the temporal resolution limitations of our design (due to time-frequency trade-off), fully disentangling these processes remains challenging. Therefore, we chose not to overemphasize this and have reported these new results in the supplementary materials. We believe that future studies, particularly those that manipulate anticipatory states or employ more suitable experimental designs, will provide

greater clarity on whether pre-stimulus activity reflects general memory-promoting state, or it is specifically linked to cognitive processes that are related to memory formation, or represents a combination that changes as a function of learning progress as suggested by our study.

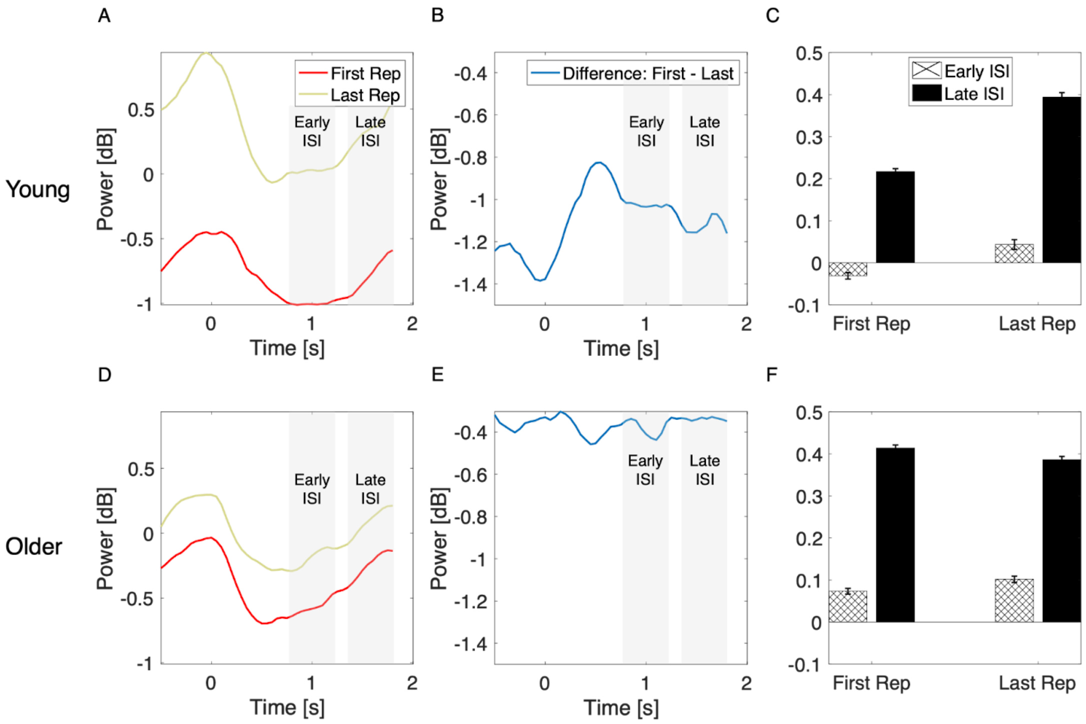

Supplementary Figure 9. Temporal evolution of parietal alpha power over stimulus presentation period and inter-stimulus-interval in young (A-C) and older (D-F) participants. Panels B and E show the difference between the first and the last (i.e., third correct recall for a given subject and sequence) sequence repetition. Panels C and F show modulation of power in early and late inter-stimulus-interval specific to ISI.

1.9. SpecParam algorithm and aperiodic adjusted power

The SpecParam algorithm (Donoghue et al., 2020) was employed to parameterize the power spectrum, effectively separating oscillatory components from the aperiodic signal. This algorithm estimates oscillatory peaks that are overlaid on the aperiodic signal, and thus, these peaks are measured relative to the aperiodic signal rather than absolute zero. The parameterization of the Power Spectral Density (PSD) is achieved through an iterative process, wherein the algorithm fits the aperiodic signal represented by L to the observed smoothed spectral signal, resulting in two parameters: the aperiodic intercept b and the

aperiodic exponent x (i.e., negative slope, with smaller x indicating a flatter spectrum). Mathematically, it can be expressed as:

$$L = b - log(k + F^x)$$

Here, F represents the vector of input frequencies, and k is the "knee" parameter, which, in this context, is set to 0 in the proposed analysis. This means that no bend of the aperiodic component is additionally modeled in the data, adhering to the default state of the SpecParam algorithm.

To extract oscillatory components, this aperiodic signal is subtracted from the power spectrum. Gaussians are then iteratively fitted to the remaining signal, and they are subtracted whenever data points exceed two standard deviations of the data. These fitted Gaussians represent the true oscillatory components in the data, while data points below the specified threshold are considered as noise. Consequently, this process yields a data-driven number of Gaussians, each characterized by the frequency center, power relative to the aperiodic signal, and the frequency bandwidth. The power spectrum is, therefore, modeled as follows:

$$P = L + \sum_{n=0}^{N} G_n + \varepsilon$$

Here, G_n represents the n_{th} Gaussian, and ε accounts for the noise not captured by the model. It's worth noting that this description of the algorithm is a simplification, and for a more detailed definition, you can refer to the original publication (Donoghue et al., 2020). The specParam algorithm was applied within a frequency range of 1 to 40 Hz, chosen to avoid overfitting of noise as small bandwidth peaks in very low frequencies. The algorithm settings included: peak width limits set to [1, 8]; no limit on the maximum number of peaks; a minimum peak height set at 0; a peak threshold defined as 2 standard deviations above the mean; and the aperiodic mode set to ‘fixed’.

Finally, to adjust the power measurements for the aperiodic signal, the aperiodic component was first reconstructed using its parameters. This reconstructed aperiodic signal was then subtracted from the total power spectrum, yielding an aperiodic-adjusted power spectrum. The aperiodic adjusted power spectrum then was used for further analysis. The extracted aperiodic component was not the focus of this work and was only briefly described in the supplementary material (Supplementary Material 5.7).

Due to the sliding window approach, spectral analysis yielded specParam model fits (i.e., R^2) ranging from -500 to 900 ms around stimulus onset, in 50 ms intervals, for each channel and sequence repetition. To compute the average specParam model fit for each sequence repetition, we averaged the fits across the time points and channels, resulting in one specParam model fit per sequence repetition. Out of 6423 sequence repetitions across all subjects, 391 (6.1%) showed an average specParam model fit below 0.9 and were excluded from further analysis (39 repetitions in young subjects and 352 repetitions in older subjects). The remaining sequence repetitions demonstrated a high overall specParam model fit, both in young (M = 0.9464, SD = 0.0127) and older (M = 0.9355, SD = 0.0157) subjects.
